# Using Skew-Logistic Probability Density Function as a Model for Age-Specific Fertility Rate Pattern

**DOI:** 10.1155/2014/790294

**Published:** 2014-05-21

**Authors:** Sahar Asili, Sadegh Rezaei, Lotfollah Najjar

**Affiliations:** ^1^Department of Statistics, Amirkabir University of Technology, Tehran 15875-4413, Iran; ^2^College of Information Science Technology, University of Nebraska-Omaha, Omaha, NE 68182, USA

## Abstract

Fertility rate is one of the most important global indexes. Past researchers found models which fit to age-specific fertility rates. For example, mixture probability density functions have been proposed for situations with bi-modal fertility patterns. This model is less useful for unimodal age-specific fertility rate patterns, so a model based on skew-symmetric (skew-normal) pdf was proposed by Mazzuco and Scarpa (2011) which was flexible for unimodal and bimodal fertility patterns. In this paper, we introduce skew-logistic probability density function as a better model: its residuals are less than those of the skew-normal model and it can more precisely estimate the parameters of the model.

## 1. Introduction

Some countries have a bimodal shape of age-specific fertility rates that classical bell shaped models [1] cannot fit suitably. It is possible to fit these patterns by means of a mixture model. Past researchers have proposed some models for these patterns, which we briefly mention as follows.

Chandola et al. [2] have proposed a Hadwiger mixture model which has seven parameters. Peristera and Kostaki in their paper [3] have developed a model, based on normal distribution which has six parameters. Schmertmann [4] has proposed a piecewise quadratic spline function which has 13 parameters. Mazzuco and Scarpa [5] have introduced a different model based on skew-normal density function which has 4 parameters. Because of the skewness parameter, that model has been suitable for most types of fertility patterns, including bimodal fertility patterns.

In this paper, as a developed by Mazzuco and Scarpa (2011) [5], a skew-logistic model is proposed. This model is fitted to the age-specific fertility rate data, and it has been shown that the residuals of this model are less than the residuals of the skew-normal model. The researchers have determined that this model estimates the parameters more precisely.

Section 2 contains a brief review of existing models of age-specific fertility rates.  Section 3 is an introduction to our proposed model for age-specific fertility rates, based upon a skew-logistic probability density function. Fitting fertility models to real data is done in Section 4. Conclusions and future research are explained in Section 5.

## 2. Summary of Some Age-Specific Fertility Models

The general form of a fertility curve is as follows:

(1) $$\begin{array}{c} g\left(x;R,\theta_{2},\ldots ,\theta_{r}\right)=R\cdot h\left(x;\theta_{2},\ldots ,\theta_{r}\right), \end{array}$$

where *h*(*x*; *θ*_2_,…, *θ*_*r*_) is a probability density function (pdf) on the real line with *r*-1 parameters and *R* is the *r*th parameter representing the total fertility rate. In different models of fertility rate, function *h*(*x*; *θ*_2_,…, *θ*_*r*_) is also different; for example, it may be inverse Gaussian, the Gamma, the Beta, the Coale-Trussell, the Brass, or the Gompertz pdfs. Using the inverse Gaussian instead of *h*(*x*; *θ*_2_,…, *θ*_*r*_) in the Hadwiger model was useful for bimodal models. The Hadwiger function is as following:

(2) $$\begin{array}{c} g\left(x;a,b,c\right)=\frac{ab}{c}{\left(\frac{c}{x}\right)}^{3/2}\exp\left\{-b^{2}\left(\frac{c}{x}+\frac{x}{c}-2\right)\right\}. \end{array}$$

In 1999, Chandola et al. introduced the “Hadwiger mixture model” [2] which has the following formula:

(3) g(x;a,m,b1,b2,c1,c2) =mab1c1(c1x)3/2exp⁡{−b12(c1x+xc1−2)}  +(1−m)ab2c2(c2x)3/2exp⁡{−b22(c2x+xc2−2)}.

Here, 0 ≤ *m* ≤ 1 is the mixture parameter. Another model was proposed in [4] with the following formula:

(4) g(x;R,α,β,θ0,θ4,t0,t4)  =R·I(α≤x≤β)·∑k=04θk(x−tk)2.

Here, *I*(·) is the indicator function, *α* and *β* are the age limits, *t*_*k*_ are the spline knots, and also *θ*_*k*_ are the parameters. Then Peristera and Kostaki [3] proposed another model for fertility patterns based on normal mixture model which has the following formula:

(5) g(x;c1,c2,μ1,μ2,σ1,σ2) =c1exp⁡{−(x−μ1σ1)2}+c2exp⁡{−(x−μ2σ2)2}.

In [5], a new model was proposed to fit age-specific data based on skew-symmetric (skew-normal) density function. This model is flexible for almost all types of fertility patterns. We briefly explain their proposed model as follows: at the beginning, we review the skew-normal pdf, which was studied in

(6) $$\begin{array}{c} f\left(x;\xi ,\omega^{2},\alpha \right)=2\omega^{-1}\phi \left(\frac{x-\xi }{\omega }\right)\Phi \left\{\alpha \left(\frac{x-\xi }{\omega }\right)\right\}. \end{array}$$

Ma and Genton [6] showed that the skew-normal pdf is unimodal, so this model is useful for fertility patterns such as USA age-specific fertility rates (1963); compare [5]. To improve this model, Mazzuco and Scarpa proved that model (1) may be generalized using the results shown in [7–10]. There is more information about this as follows.

For any symmetric pdf *f*_0_ and distribution function *G* with a symmetric density, function (2) is a density function for any odd function *ω*(·). Consider

(7) $$\begin{array}{c} f\left(x\right)=2f_{0}\left(x\right)G\left\{\omega \left(x\right)\right\}. \end{array}$$

Now if pdf and cdf of standard normal distribution replace *f*_0_ and *G*, respectively, and insert *ω*(*x*) = *αx*, the Flexible Generalized Skew-Normal (FGSN) distributions formed as follows:

(8) f(x;ξ,ω2,α,β)  =2ω−1ϕ(x−ξω)Φ{α(x−ξω)+β(x−ξω)3}.

According to [6], the pdf in (8) has at almost two modes, so this model is adequate for bimodal fertility patterns. As you can see, there are now 4 parameters *α*, *β*, *ξ*, and *ω* in this model which will be interpreted as the following.

Parameter *ξ* is location parameter, and *ω* is scale parameter. Parameter *α* is the skewness parameter in the skew-normal distribution when *β* = 0, and, in the FGSN distribution, *β* is the skewness parameter. The two parameters *α* and *β* are related to the location of two modes fertility patterns. We do not have the exact value of them, so we assumed that they vary between −5 and +5. According to Mazzuco and Scarpa, there is different plots for the situations in which *ξ*, *ω*, and *α* are fixed, and the parameter *β* has various values. After interpreting the parameters of Mazzuco and Scarpa [5], their model was fitted to real data. Notice that parameters of fertility patterns are estimated through nonlinear least squares, by minimizing the following term:

(9) $$\begin{array}{c} S\left(R,\theta_{2},\ldots ,\theta_{r}\right)=\overset{e}{\underset{x=b}{\sum }}{\left\{g\left(x;R,\theta_{2},\ldots ,\theta_{r}\right)-f_{x}\right\}}^{2}, \end{array}$$

where *f*_*x*_ is the real age-specific fertility rate, *g*(*x*; *R*, *θ*_2_,…, *θ*_*r*_) is the fertility rate at age *x* given by the fertility model used, and *b* and *e* are the ages at the beginning and at the end of the fertile period, respectively; compare [5]. In that paper, you can find two figures, the Italian fertility model and the USA fertility model. In these figures, the residuals of the skew-normal model are somewhat less than those of the other models. Although there are some models in these figures which do not have smaller residuals, because these models have more parameters, again we can claim that skew-normal is preferred.

In the next section, we show that the skew-logistic model as a new fertility model has 4 parameters, just as the skew-normal model. However, its residuals are less than skew-normal, so it is the preferred model so far.

## 3. Skew-Logistic Distribution as a Fertility Model

In this paper, our aim is to introduce and to fit a skew-logistic model for age-specific fertility patterns.

Equations (10) and (11) show cdf and pdf of the logistic respectively:

(10) $$\begin{array}{c} G\left(x\right)=\frac{1}{1+e^{-\lambda x}}, \end{array}$$

(11) $$\begin{array}{c} g\left(x\right)=\frac{\lambda e^{-\lambda x}}{{\left(1+e^{-\lambda x}\right)}^{2}}. \end{array}$$

The following formula was introduced by Azzalini for skewing a symmetric distribution:

(12) $$\begin{array}{c} f\left(x\right)=2f_{0}\left(x\right)G\left\{\omega \left(x\right)\right\}. \end{array}$$

If we replace (10) and (11) instead of *G* and *f*_0_ at (12) and put *ω* = *αx*, we will have skew-logistic distribution function which is as follows:

(13) $$\begin{array}{c} f_{\text{sl}}\left(x;\alpha \right)=\frac{2e^{-x}}{{\left(1+e^{-x}\right)}^{2}\left(1+e^{-\alpha x}\right)}. \end{array}$$

Here, *α* is the skewness parameter and *x* ∈ *R*. Now we transform *x* to (*y* − *μ*)/*σ*, in which *μ* is the location parameter and *σ* is the scale parameter:

(14) fsl(y;α,μ,σ)=2e−(y−μ)/σσ(1+e−(y−μ)/σ)2(1+e−α((y−μ)/σ));                       y∈R.

If we rewrite (14) based on the odd power, we will have

(15) fsl(y;α,β,μ,σ) =2σ−1·e−(y−μ)/σ(1+e−(y−μ)/σ)2(1+e−α((y−μ)/σ)−β((y−μ)/σ)3).

**Figure 1:**
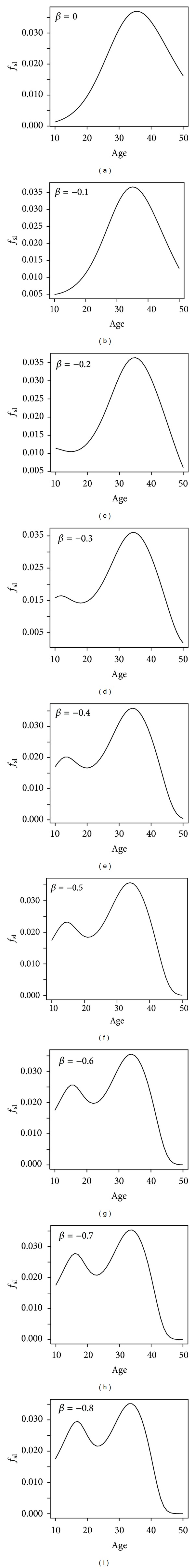
Skew-logistic probability function when *ξ* = 30, *σ* = 8, *α* = 1, and *β* has different value.

Equation (15) is the model which we propose for age-specific fertility patterns. Examples which show that this pdf can have two modes are in Figure 1.

## 4. Fitting Fertility Models to Real Data

**Figure 2:**
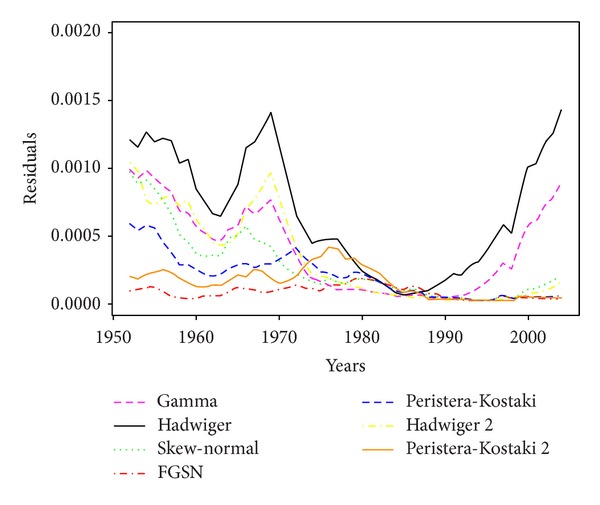
Residuals of fertility models fitted to Italy data.

As it is shown in [5] and Figure 2, which is for an Italian data set, the skew-normal model is generally a better fit, with lower residuals.

In this section, the skew-normal and skew-logistic fertility models will be fitted to real data and their quality will be assessed. The results show that the skew-logistic's fit is better than that of skew-normal, so, with respect to Mazzuco and Scarpa's findings, we conclude that skew-logistic has the best fit of the models mentioned above.

**Figure 3:**
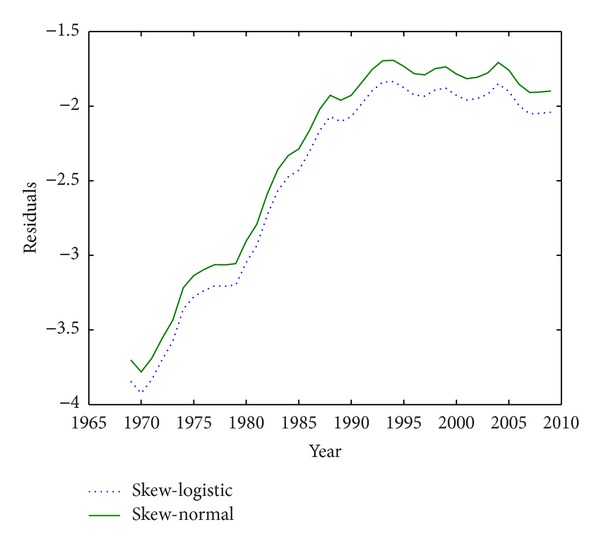
Residuals of fertility models fitted to Ireland data.

**Figure 4:**
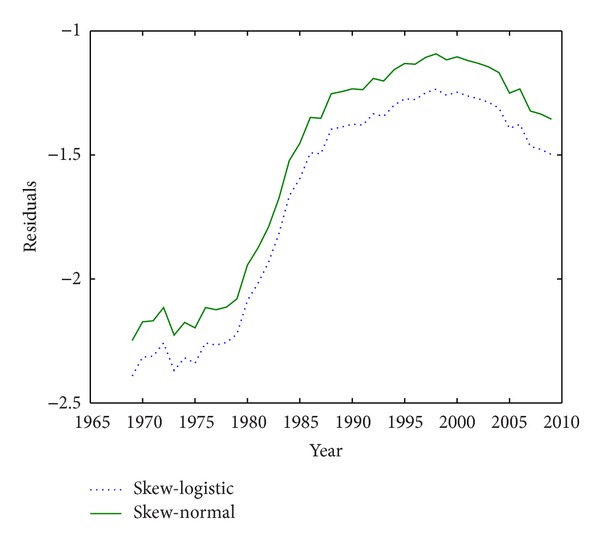
Residuals of fertility models fitted to Greece data.

To compare, we will use the fertility data of Ireland in the same years. The data of Greece will be surveyed in Figure 3. Data are taken from Human Fertility Database (HFD) [11] and Eurostat (http://epp.eurostat.ec.europa.eu/). Notice that the parameters of the model will be estimated through nonlinear least squares, just as skew-normal; it means by minimizing (9) that here *g*(*x*; *R*, *θ*_2_,…, *θ*_*r*_) is the fertility rate at age *x* given by the skew-logistic fertility model. This model is fitted to the age-specific fertility rate data of Ireland in Figure 3. Looking at this figure, it appears that skew-normal and skew-logistic have a similar pattern but it is clear that skew-logistic is preferred to skew-normal because its sum of square of residuals is much lower than that of skew-normal. It is also superior to other models introduced later (there is more information in Section 5). Also, fitting the model to age-specific fertility rate data of Greece in Figure 4, it is again shown that the skew-logistic has a lower sum of square of residuals.

## 5. Conclusions

A new fertility model has been proposed which is based on skew-logistic probability density function. Previously, other models were used which had multiple parameters and so were less ideal for our aim. Recently, Mazzuco and Scarpa (2011) [5] proposed a model based on generalization of the skew-normal distribution (FGSN). The advantage of the FGSN model is its flexibility for complex fertility patterns and also the number of its parameters, which is lower than the later models. We showed that the model based on the skew-logistic distribution function is better than skew-normal not because of having lower number of parameters but because of having a better fit to data. For future research, it is proposed that student's *t*-distribution is used instead of logistic.

Because it may be possible that a model based on student's *t*-distribution will have fewer parameters. It is also possible that, skewing by Fernndez and Steel [12] method instead of Azzalini [13], better fit to real data may be produced.

Finally it is necessary to discover whether the MATLAB program could be used for calculating parameters and drawing graphs. Outputs of this program show that, with the same data, the norm of the residual (resnorm) of the skew-normal model is 148.7917 and the resnorm of the skew-logistic model is 143.5909. This shows that the accuracy of the latter is greater than the former.
